# Treatment of Peritonsillar Abscess in Children: A Systematic Review

**DOI:** 10.3390/jcm13237361

**Published:** 2024-12-03

**Authors:** Francesca Galluzzi, Werner Garavello

**Affiliations:** 1 Department of Otorhinolaryngology, Fondazione IRCCS San Gerardo dei Tintori, 20900 Monza, Italy; werner.garavello@unimib.it; 2 Department of Otorhinolaryngology, School of Medicine and Surgery, University of Milano-Bicocca, 20126 Milan, Italy

**Keywords:** peritonsillar abscess, children, treatment, surgery, review

## Abstract

**Objectives:** This review aims to analyze the treatment options for peritonsillar abscess (PTA) in children. **Methods:** We searched PubMed and EMBASE for studies regarding the treatment of PTA in children. Relevant studies were reviewed based on systematic review (PRISMA) guidelines. Qualitative and quantitative analyses of the extracted data were performed. **Results:** A total of 12 articles with 2211 cases of PTA were found to be eligible. All the identified studies were retrospective cohorts. The mean age varied from 8.5 to 15.4 years without gender difference. Treatment options included broad-spectrum antibiotic therapy with incision and drainage of the abscess, needle aspiration and immediate tonsillectomy in 69%, 7.6% and 7% of cases, respectively. Antibiotics alone were administered to 16.4% of the subjects. The rate of recurrence of PTA after primary treatment ranged from 2% to 15.8% of cases. The time of recurrence is considered within one or two months. Complications in children with PTA were torticollis, prolonged fever, sepsis, dyspnea and parapharyngeal involvement. **Conclusions:** The mainstay of treatment of PTA in children is antibiotic therapy with incision and drainage of the abscess. Alternatives include antibiotic treatment alone or in association with needle aspiration. Immediate tonsillectomy is reserved only for a few high-risk patients.

## 1. Introduction

Peritonsillar abscess (PTA) is the most common deep-neck infection in children [1]. It is defined as a collection of pus between the tonsillar capsule, superior constrictor muscle and the palatopharyngeus muscles. The usual course of developing PTA may result from the suppuration of soft tissue due to acute tonsillitis or pharyngitis. Infrequently, the obstruction of Weber’s gland located in the superior tonsillar pole may be the inciting factor [2]. In the vast majority of cases, PTA is unilateral and most commonly affects adolescents [1].

The infection often is polymicrobial, including aerobes and anaerobes. The most frequently cultured pathogens are Streptococcus pyogenes [3]. Although recurrent tonsillitis has been considered a risk factor for PTA, an association has not yet been demonstrated [4]. Indeed, it is still unclear why in the pediatric population there is a low incidence of PTA in the face of a high prevalence of tonsillitis [5].

The diagnosis is based on history, clinical evaluation and laboratory tests. In uncooperative children or in case of suspicion of extended abscess in the parapharyngeal space, an additional computed tomography (CT) scan may be needed. Contrast-enhanced CT typically shows PTA as an irregularly shaped fluid collection involving the palatine tonsil with extension into parapharyngeal soft tissues [6].

Transcutaneous ultrasound can differentiate PTA from other tonsillar infections; therefore, it has been considered useful in identifying patients who do not require surgery [7,8]. The most definitive diagnostic procedure for PTA is needle aspiration, which can provide microbiological analysis and some drainage of the abscess. Surgical treatments consist of the incision and drainage (I&D) of the abscess that can be performed in local or general anesthesia depending on the age and cooperation of the patient and, in selected patients, the immediate tonsillectomy. Because awake procedures can lead to suboptimal outcomes and potentially create distress for both the child and parents in uncooperative children, conscious sedation has emerged as a promising option to ensure safety and improve the efficacy of treatment.

Medical therapy involves hydration, pain control and antibiotics, such as amoxicillin and clavulanate, clindamycin and cefotaxime or piperacillin and tazobactam [9], which can be adjusted accordingly as bacterial sensitivities obtained from abscess culture. Corticosteroids can also be used to reduce the inflammation and edema of oropharynx and improve pain control, although their application is still controversial [2].

Untreated or neglected PTA can result in serious complications due to the spread of the infection, such as airway compromise, aspiration pneumonia, mediastinitis, sepsis and jugular vein thrombosis [10].

There is still controversy surrounding the management of PTA in children with different proposed protocols [5,11], and a systematic literature review is lacking.

We perform this systematic review focused on the treatment of PTA in children in order to identify the current medical and surgical trends.

## 2. Materials and Methods

### 2.1. Search Strategy

A systematic review of the literature was completed. A search of the literature was conducted in accordance with the Preferred Reporting Items for Systematic Reviews and Meta-analyses (PRISMA) guidelines [12]. PubMed and EMBASE databases were systematically searched through July 2024. Searches were conducted by combining the following terms (“peritonsillar abscess”) AND (“children” OR “pediatric”) AND (“treatment” OR “therapy”). Bibliographies were searched for additional articles. The last search was performed on 30 September 2024. The review protocol was registered in the International Prospective Register of Systematic Reviews (PROSPERO; registration number: CRD42022302437).

### 2.2. Study Selection

Study eligibility criteria were applied independently by two authors (F.G. and W.G.). Any disagreements were resolved by consensus. Articles were selected in two phases: first, titles and abstracts were screened for those apparently meeting inclusion criteria, and then the full texts of selected articles were read, excluding those that did not meet the review eligibility criteria.

Inclusion criteria: (1) studies regarding the treatment of peritonsillar abscess in children; (2) studies published from 1990 in peer-reviewed journals; (3) English language. Exclusion criteria: (1) reviews, meta-analyses, case reports, editorials, letters, meeting abstracts, personal comments or book chapters; (2) studies considering adult population; (3) studies reporting incomplete data.

### 2.3. Data Extraction

Data extraction from the included studies was performed and independently verified by F.G. and W.G.

A standardized full-text analysis was performed, and data were gathered on the main characteristics of the studies (first author, year of publication, type of study, number of cases of PTA, age, sex, symptoms, signs, microbiological findings) and concerning the type of treatment (needle aspiration, I&D, tonsillectomy, antibiotics), recurrence (number and timing), complications and duration of follow-up.

### 2.4. Quality of Evidence Assessment

The Joanna Briggs Institute (JBI) critical appraisal checklist for case series assessment tools was used to appraise the quality of the studies (Table S1). Each item was rated yes, no or unclear. A “yes” response was scored 1 point, and “no” and “unclear” responses were scored 0 points. For each case series, “Good” was defined as at least seven out of ten criteria met, “Fair” as five or six criteria met and “Poor” as four or fewer criteria met (Table S2). The average score for each article was independently assessed by two authors (F.G. and G.W.), and discrepancies were resolved by consensus [13]. Overall, only studies with a “good” rating on the JBI critical appraisal checklist were included (Table S2).

### 2.5. Data Synthesis

We performed qualitative and quantitative analyses of the extracted data. A meta-analysis providing statistical evidence was not achieved due to the heterogenicity and limited data of the included studies.

## 3. Results

**Figure 1:**
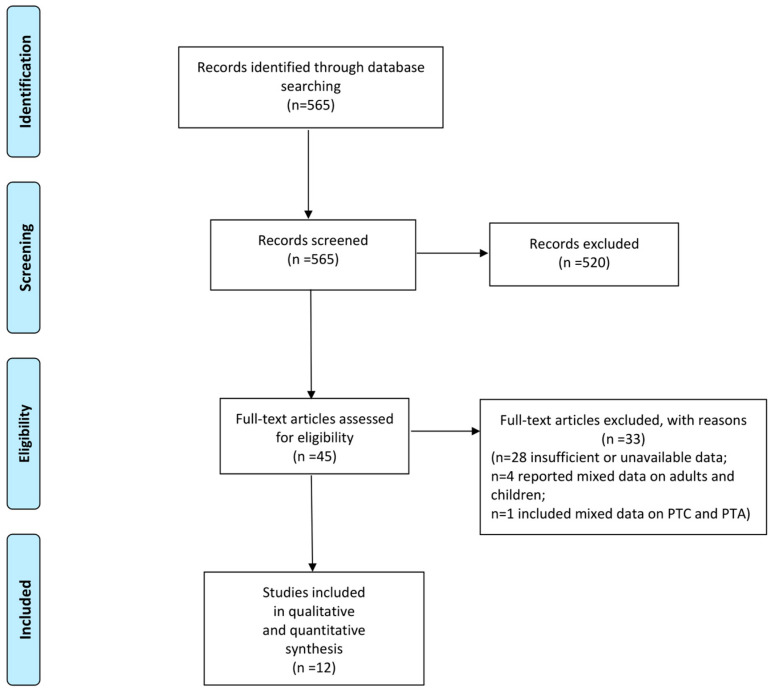
PRISMA flowchart for selection of studies [12].

Following a systematic review, 12 articles were finally included. The PRISMA flow diagram is shown in Figure 1.

All the included studies were retrospective. A total of 2211 children with PTA were included. The sample sizes among the studies ranged from 19 [14] to 777 [15]. As detailed in Table 1, the mean age varied from 8.5 [16] to 15.4 [17] years and the male-to-female ratio was 1:1 [4,15,16,18,19,20,21,22]. Seven studies reported clinical and microbiological data [11,14,16,17,18,19,23]. All patients complained of sore throat with or without drooling, fever, odynophagia, muffled voice and neck pain. The main oropharyngeal sign was a unilateral peritonsillar bulge with uvular deviation associated with trismus and neck adenopathy. Two authors described compromised airways [17] and dehydration [11]. The most frequent isolated bacteria was beta-hemolytic Streptococcus group A and mixed flora with or without anaerobes.

Treatment modalities, recurrences and complications are described in Table 2. I&D, needle aspiration and immediate tonsillectomy were performed in 1372 [4,11,14,15,18,19,20,21,22], 153 [4,14,19,23] and 139 [4,11,14,15,16,20,23] subjects, respectively. Antibiotics alone were administrated to 328 children [11,16,19,20,23]. Three authors did not specify the surgical treatment modalities [16,17,20] and only two authors reported the type of antibiotic therapy [4,20].

**Figure 2:**
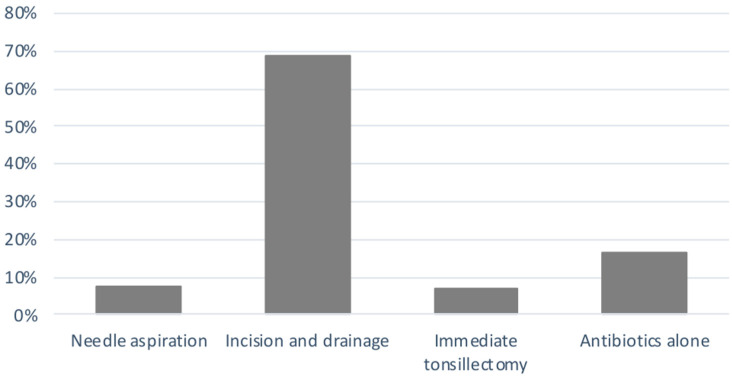
Treatment modalities for PTA in children.

The percentage of different types of surgical treatment modalities for PTA is shown in Figure 2.

Seven authors reported the rate of recurrence of PTA after primary treatment [11,14,15,17,18,20,21] ranging from 2% [20] to 15.8% [18] in cases depending on the studies. The time of recurrence is considered within one [18,21] or two months [17].

Four authors described the following complications in children with PTA: torticollis, prolonged fever, sepsis, dyspnea and parapharyngealinvolvement [14,15,18,20]. Only three authors specified the duration of follow-up, which ranged from one month [15,18] to two years [14].

## 4. Discussion

As a result of this review, in the evaluation of treatment options for pediatric PTA, antibiotic therapy associated with the I&D of an abscess was the modality of choice (69% of cases) (Figure 2). This procedure involves an incision in the mucosa superior to the tonsil and the drainage of the abscess. Due to the inability of children to cooperate, this procedure may require treatment in the operating room in up to half of cases [11]. However, especially in older children, it can be performed in a controlled setting such as the emergency department with proper equipment [2,18,24]. In this regard, Ghantous et al. studied 118 children undergoing I&D for PTA under local anesthesia (103 episodes), under conscious sedation (42 episodes) and ten treated with IV antibiotics only. They found that conscious sedation facilitates PTA drainage with excellent safety and improved efficacy compared to local anesthesia. Indeed, the amount of pus drained from the abscess was higher with conscious sedation when compared to non-conscious sedation (4.9 ± 4 mL vs. 3.2 ± 2 mL, *p* = 0.03), the maximum pain scores were lower with conscious sedation than non-conscious sedation (1.4 ± 2 vs. 4.2 ± 3, *p* < 0.001) and only one minor oxygen desaturation (2%) event occurred [24]. Although this is a valid option in selected children, there are some crucial points to consider: (1) it requires specialized personnel trained in pediatric sedation, which may not be available in all hospitals; (2) it carries potential risks of complications related to sedation, especially if the airways are compromised; (3) it requires potentially longer procedure times and increased resource utilization.

Considering the other treatment options, we found that antibiotics alone, needle aspiration and tonsillectomy were performed in 16.4%, 7.6% and 7% of cases, respectively (Figure 2). Supporting our results, a US national survey including 20,546 subjects found a significant increase in the rate of I&D from 26.4% to 33.7% (*p* < 0.001) and a significant decrease in the rate of tonsillectomy from 13.8% to 7.8% (*p* < 0.001) [25]. Contrarily, Nguyen et al., in a retrospective study including 2994 patients who presented with peritonsillar cellulitis or abscess, found that the most common treatment of choice was medical therapy alone (30.8%), followed by I&D (30.5%) and tonsillectomy (9.4%) [26].

Antibiotic therapy alone is a valid option and, as reported by Kim et al., the predictive factors of its efficacy were younger patients, fewer episodes of acute tonsillitis and smaller abscess size [16]. However, Hsiao et al., comparing children with PTA receiving surgical (n = 48) and non-surgical procedures (n = 8), found that children with antibiotic treatment alone were younger and had a longer duration of hospital stay (5.7 vs. 8.1 *p* < 0.001) [20]. To date, there are no studies that compare the effectiveness of different types of antibiotics used as the sole therapy.

Needle aspiration as a treatment modality for PTA appears to be an efficacious and safe method [19,23]. Weinberg et al. treated 41 children and resolved the infection in 87% of cases. Two patients needed to repeat aspiration (6%) and two underwent immediate tonsillectomy (6%) for persistent abscess. No complications were described [23]. Though injury of the internal carotid artery (ICA) is a feared complication during needle aspiration, it has never been reported in the medical literature. Some authors advocated for the use of transoral ultrasound, which helps visualize important neighboring structures such as ICA and avoid this possible adverse event [27].

Immediate tonsillectomy, also known as quinsy tonsillectomy, is rarely indicated. It allows for the complete drainage of an abscess and avoids any recurrence. Controversy over its application remains due to the unclear balance between surgical risks and benefits [26,27,28]. Most authors suggest performing immediate tonsillectomy in case of failure of I&D, recurrent PTA, recurrent tonsillitis and obstructive sleep apnea symptoms [11,14,18]. However, recently, Rosi-Schumacher et al., studying 777 children treated for PTA, found that for quinsy tonsillectomy versus I&D, there was no statistically significant difference in length of stay (1.9 vs. 1.7 days, *p* = 0.523), readmission (17 vs. 0, *p* = 0.265) or return to the operating room (18 vs. 1, *p* = 0.810). Therefore, they considered quinsy tonsillectomy to be a good option in cases of PTA or recurrent tonsillitis [15].

**Figure 3:**
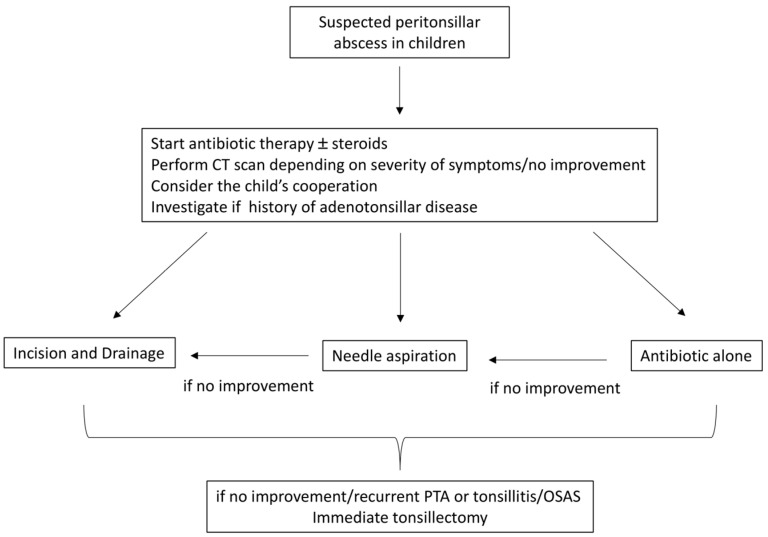
Treatment algorithm for PTA in children.

In order to improve the decision-making process for the treatment of PTA in children, we propose an algorithm that shows the different therapeutic approaches (Figure 3). As previously described by Schraff et al., the treatment decision is based on the level of cooperativeness of the child and previous history of adenotonsillar disease [11].

Few authors have studied the recurrence rate of PTA, reporting a range between 2% [20] to 15.8% [18], with time of recurrence within one [18,21] or two months [17]. Allen et al., studying 566 cases of pediatric PTA, found that recurrence was associated with older age (*p* = 0.005) and a history of recurrent tonsillitis (*p* < 0.0001). They also noted that there was not a statistically significant difference in terms of recurrence for patients managed in an outpatient over an inpatient setting [21]. Furthermore, Ghantous et al. found that the recurrence rate in children undergoing I&D with conscious sedation was numerically lower than in the non-conscious sedation group (5% vs. 14%), although this difference did not reach statistical significance [24].

Chang BA et al., in a systematic review including 674 participants (adults and children), compared the effectiveness of needle aspiration and I&D for the treatment of PTA. They found that very low-quality evidence suggested that I&D may be associated with a lower chance of recurrence than needle aspiration and that there was very low-quality evidence to suggest that needle aspiration was less painful [29].

Considering the characteristics of the population studied, we found that PTA affects mainly adolescents without a gender difference. Typical symptoms (sore throat, fever, odynophagia, muffled voice and neck pain) and signs (unilateral peritonsillar bulge with uvular deviation, trismus and neck adenopathy) allow for PTA diagnosis (Table 1) even if some difficulties may be present in younger or uncooperative subjects [5]. Complications are described in cases of severe abscess and untreated or non-respondent patients [14,18,20]. Though rare, compromised airways and parapharyngeal involvement are the most critical conditions that may require urgent management.

As detailed in Table 1, the microbiological aspect of PTA revealed that beta-hemolytic Streptococcus group A and mixed flora with or without anaerobes were the most frequently isolated germs. To date, there is no consensus on appropriate antibiotic therapy, and most recommendations are based on studies on antibiotic sensitivities of microorganisms identified in the purulent secretion of PTA, suggesting initial empiric therapy with a broad-spectrum antibiotic such as penicillin or amoxicillin/ac clavulanate with metronidazole [3]. Only two authors of the included studies reported the types of antibiotics used [4,20]; therefore, no conclusions could be drawn.

Therefore, considering the low level of the reviewed evidence, further studies comparing the different treatment options (needle aspiration, I&D, antibiotic therapy alone and immediate tonsillectomy) are needed to ensure the best possible care for children with PTA, especially in this era of increased antimicrobial resistance.

This review has some limitations. One, it includes only retrospective studies with a wide range of samples. Two, some of the included studies have incomplete data such as the evaluation of recurrences and the duration of follow-up. Three, only one of the included retrospective studies compared groups of children undergoing different treatment options.

## 5. Conclusions

Treatment of PTA in children is currently considered antibiotic therapy with the I&D of the abscess. Antibiotics alone or combined with needle aspiration may be a valid alternative option in selected patients. Rarely, immediate tonsillectomy can help children with a high risk of recurrence or airway obstruction. Future studies comparing the different treatment options may allow us to define and validate protocol for the management of pediatric PTA.

## Figures and Tables

**Table 1:** Characteristics of the included studies.

Authors, Year	Study	N° Cases PTA	Mean Age (y)	Sex	Symptoms PTA	Signs	Microbiology
Weinberg et al., 1993 [23]	Retrospective	43	13.9	-	Sore throat 100% Drooling 34% Muffled voice 59%	Unilateral peritonsillar bulge 100% Trismus	GABHS, Str. Viridans Fusobacterium necrophorum H. influenzae
Apostolopoulos et al., 1995 [18]	Retrospective	189	9	86M:103F	Sore throat	Peritonsillar bulge Trismus	GABHS 35% Anaerobes 12% Others 12% Str. Viridans 10% Str. non-A 7% St. Aureus 6% Candida 6% H. Influenzae 4.5% Str. pneumoniae 4.5% Str. Sanguis 3%
Wolf et al., 1995 [14]	Retrospective	19	10–16	-	Pain and dysphagia	Trismus 50% Fever	GABHS St. Aureus Str. Viridans Str. Non-A Pneumococci Peptostreptococci Mixed flora
Schraff et al., 2001 [11]	Retrospective	83	12.1	-	Sore throat/neck pain 93% Odynophagia 83% Muffled voice 37%	Neck adenopathy 94% Uvular deviation 52% Trismus 30% Dehydration 47% Fever 55%	Mixed flora with Str. pyogenes the predominant organism
Millar et al., 2007 [17]	Retrospective	43 PTA 178 PTC	15.4 PTA 3.2 PTC	-	Sore throat 100% Painful swallowing 100% Voice changes 86.7% Decreased oral intake 90.6% Drooling 75%	Peritonsillar swelling 100% Cervical adenopathy 96.1% Trismus 78.9% Uvular deviation 73.3% Airway compromise 8% Fever 59.5%	GABHS Str. non-group A St. Aureus
Segal et al., 2009 [4]	Retrospective	126	12.8	55M:71F	-	-	GABHS 45.3% Anaerobes 14% Mixed w/o anaerobes 15.6% Str. C 6.2% others 17.3
Chang et al., 2010 [19]	Retrospective	21	14.8	10M:11F	Odynophagia 21%	Fever 61.9% Trismus 4% Uvular deviation 6% Neck pain/mass 1%	Mixed flora
Hsiao et al., 2012 [20]	Retrospective	56	12.9	31M:24F	Sore throat	Fever Asymmetric Swollen/bulging tonsil Uvular deviation	Str. 72% Fusobacterium species 44% Anaerobes 74%
Kim et al., 2015 [16]	Retrospective	88	8.5	52M:36F	-	-	-
Allen et al., 2019 [21]	Retrospective	566	12.9 outpt 9.9 inpt	261M:305F	-	-	-
Chisholm et al., 2020 [22]	Retrospective	200	12.6	77M:123F	-	-	-
Rosi-Schumacher et al., 2023 [15]	Retrospective	777	10.7	357M:420F	Sepsis 45.9% Systemic inflammatory response syndrome 4.8%	-	-

PTA: peritonsillar abscess; PTC: peritonsillar cellulitis; GABHS: beta-hemolytic Streptococcus group A; Str: streptococcus; St: staphylococcus m: month; y: year; outpt: outpatient; inpt: inpatient.

**Table 2:** Types of treatments for pediatric PTA.

Authors, Year	Needle Aspiration and/or Incision and Drainage	Tonsillectomy	Antibiotics	Recurrence (%)	Time of Recurrence	Complications	Follow-Up
Weinberg et al., 1993 [23]	41 needle aspiration (31 positive, 10 negative)	5 immediate	7 antibiotics alone	-	-	-	-
Apostolopoulos et al., 1995 [18]	136 I&D (53 negative)	-	-	15.8%	1 m	12 (6.3%) torticollis, prologed fever	1 m–7 y
Wolf et al., 1995 [14]	7 needle aspiration (6 LA, 1 GA) 12 I&D (5 LA, 7 GA)	2 immediate 1 elective	17 antibiotics initiated 1–8 d prior the admission	0	-	1 torticollis 1 dyspnea	2 y
Schraff et al., 2001 [11]	54 I&D	25 immediate	3 antibiotics alone	0	-	-	-
Millar et al., 2007 [17]	43 needle aspiration or I&D	-	-	4.7%	Within 60 d	-	-
Segal et al., 2009 [4]	95 needle aspiration 30 I&D GA	1 immediate	64.2% amoxicillin-clavulanate, 19% cefuroxime 13.5% cefuroxime + metronidazole 2.1% azithromycin	-	-	-	-
Chang et al., 2010 [19]	3 I&D 10 needle aspiration	-	8 antibiotics alone	-	-	No complications	-
Hsiao et al., 2012 [20]	48	1 elective	9 penic. 15 penic. + genta. 4 penic. + clyndamicina 5 penic. + clyndamicina + genta 12 amox.cl. 5 amox.cl + genta. 1 amox.cl + ciprofloxacina 3 ampicillina/sulbactam 1 oxacillin + genta. 1 vancomycin + ceftazidime 8 intravenous antibiotics alone	2%		1 IOT 2 parapharyngeal involvement	
Kim et al., 2015 [16]	55 any surgery “Poor responder”	0	33 antibiotic alone “good responder”	-	-	-	-
Allen et al., 2019 [21]	113 I&D outpt 184 I&D inpt	Immediate 12 outpt + 42 inpt elective 22 outpt + 33 inpt	antibiotics only 181 outpt + 88 inpt	9.1% 29 outpt 23 inpt	Within 30 d	-	-
Chisholm et al., 2020 [22]	115 I&D	-	-	-	-	-	-
Rosi-Schumacher et al., 2023 [15]	725 I&D	52 immediate 6 elective	-	2.5%	-	357 sepsis 37 systemic inflammatory response syndrome	1 m

LA: local anesthesia, GA: general anesthesia; d: days; m:month; y: year; I&D: Incision and Drainage; outpt: outpatients; inpt: inpatient: amox.cl.: amoxicillin + clavulanic acid; penic: penicillin; genta: gentamicin.

## Data Availability

Data extracted from the included articles are available in PubMed and EMBASE databases.

## Supplementary Materials

The following supporting information can be downloaded at: https://www.mdpi.com/article/10.3390/jcm13237361/s1, Table S1: The Joanna Briggs Institute Critical Appraisal Checklist; Table S2. The JBI Critical Appraisal Checklist of the included studies.
