# Supplementary material for: Treatment of Peritonsillar Abscess in Children: A Systematic Review
Source: J Clin Med. 2024 Dec 3;13(23):7361. doi: 10.3390/jcm13237361 (PMC11642394; doi:10.3390/jcm13237361)
Supplement: Supplementary file 1 [file jcm-13-07361-s001.zip › jcm-3322527-supplementary.pdf]

Table S1. The Joanna Briggs Institute Critical Appraisal Checklist

Questions	Yes	No	Unclear	Not applicable
1. Were clear criteria for inclusion in the case series?				
2. Was the condition measured in a standard, reliable way for all participants included in the case series?				
3. Were valid methods used for identification of condition for all participants included in the case series?				
4. Did the case series have consecutive inclusion of participants?				
5. Did the case series have complete inclusion of participants?				
6. Was there clear reporting of demographics of participants in the study?				
7. Was there clear reporting of clinical information of participants?				
8. Were the outcomes or follow-up results of cases clearly reported?				
9. Was there clear reporting of presenting sites'/clinics' demographic information?				
10. Was statistical analysis appropriate?				

Table S2. The JBI Critical Appraisal Checklist of the included studies

Authors, years	Q1	Q2	Q3	Q4	Q5	Q6	Q7	Q8	Q9	Q10	Overall rating
Weinberg et al.,1993 [23]	yes	yes	yes	yes	yes	un	yes	yes	no	N/A	7
Apostolopoulos et al., 1995 [18]	yes	yes	yes	yes	yes	yes	yes	yes	yes	N/A	8
Wolf et al., 1995 [14]	yes	yes	yes	yes	yes	un	yes	yes	no	N/A	7
Schraff et al., 2001 [11]	yes	yes	yes	yes	yes	un	yes	yes	no	N/A	7
Millar et al., 2007 [17]	yes	yes	yes	yes	yes	yes	yes	yes	yes	yes	10
Segal et al., 2009 [4]	yes	yes	yes	yes	yes	un	yes	un	yes	N/A	7
Chang et al., 2010 [19]	yes	yes	yes	yes	yes	yes	yes	un	yes	yes	9
Hsiao et al., 2012 [20]	yes	yes	yes	yes	yes	yes	yes	yes	yes	yes	10
Kim et al., 2015 [16]	yes	yes	yes	yes	yes	yes	yes	yes	yes	yes	10
Allen et al., 2019 [21]	yes	yes	un	yes	yes	yes	no	yes	no	yes	7
Chisholm et al., 2020 [22]	yes	yes	yes	yes	yes	yes	no	no	no	yes	7
Rosi-Schumacher et al., 2023 [15]	yes	yes	yes	yes	yes	yes	un	un	un	yes	7
